# Multi-institutional collaborative mock oral (mICMO) examination for cardiothoracic surgery trainees: Results from the pilot experience

**DOI:** 10.1016/j.xjon.2020.07.007

**Published:** 2020-07-25

**Authors:** Amy G. Fiedler, Dominic Emerson, Erin A. Gillaspie, Joshua L. Hermsen, Melissa M. Levack, Daniel P. McCarthy, Smita Sihag, Stephanie G. Worrell, Mara B. Antonoff

**Affiliations:** a Division of Cardiothoracic Surgery, Department of Surgery, University of Wisconsin, Madison, Wis; b Department of Cardiac Surgery, Cedars Sinai Medical Center, Smidt Heart Institute, Los Angeles, Calif; c Vanderbilt University Medical Center, Nashville, Tenn; d Department of Surgery, Memorial Sloan Kettering, Thoracic Service, New York, NY; e Division of Thoracic Surgery, Case Western Reserve University, Cleveland, Ohio; f Department of Thoracic and Cardiovascular Surgery, UT MD Anderson Cancer Center, Houston, Tex

**Keywords:** education, cardiothoracic surgery training, ABTS examination, mock orals, ABS, American Board of Surgery, ABTS, American Board of Thoracic Surgery, ACGME, Accreditation Council for Graduate Medical Education, CE, Certifying Examination, COVID-19, coronavirus disease 2019, mICMO, multi-institutional collaborative mock oral, MOE, mock oral examination, TECoG, Thoracic Educational Cooperative Group

## Abstract

**Objective:**

The American Board of Thoracic Surgery–certifying examination is challenging for applicants. Single institutions have reported good results with a mock oral examination (MOE) for trainees. General surgery literature has demonstrated success with in-person multi-institutional MOE examinations. Due to small numbers of cardiothoracic training programs and significant geographic variability, we hypothesized that a multi-institutional, collaborative remotely administered MOE (mICMO) pairing faculty with trainees from different institutions would provide an important educational experience.

**Methods:**

mICMOs were conducted via the Zoom virtual platform across 6 institutions. Descriptive results via post-experience survey were analyzed and reported.

**Results:**

In total, 100% of trainees found mICMO useful. The majority would recommend to a peer, and 100% of faculty examiners found mICMO useful and would participate in another examination.

**Conclusions:**

Faculty and trainees found the experience to be effective with respect to creating a high-stakes environment, educationally beneficial, and productive. These results support the continued use of mICMO and encourage expansion and collaboration with additional institutions across the country.

**Figure:**
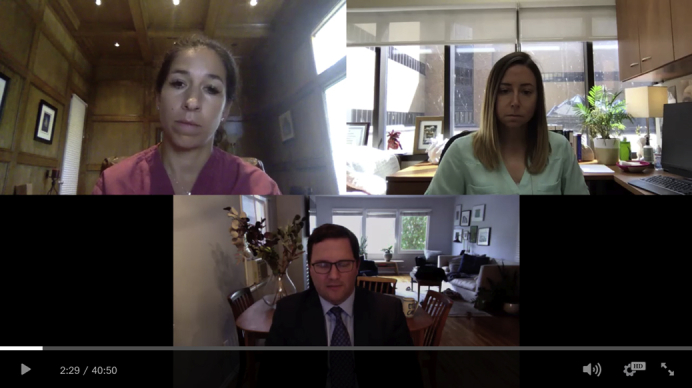
Drs Antonoff and Fiedler examine a trainee during the pilot mICMO examination.

**Central Message**

mICMO is a collaborative effort across institutions to virtually simulate a high-stakes MOE of the ABTS qualifying examination to educate and prepare our trainees for board certification.

**Perspective**

The ABTS-certifying examination is challenging for applicants. An MOE has been shown to improve trainees' performance. Creating a simulated, virtual, high-stakes MOE with trainees examined by faculty outside their home institution has not been investigated. We found that this multi-institutional collaborative effort is useful and educationally beneficial.

See Commentaries on pages 136 and 138.

Successfully preparing for, taking, and passing The American Board of Thoracic Surgery (ABTS) certifying and qualifying examinations and becoming a board-certified cardiothoracic surgeon is a challenging endeavor and a major accomplishment for graduates of Accreditation Council for Graduate Medical Education (ACGME)-accredited cardiothoracic surgery training programs.^1^, ^2^, ^3^ To become ABTS-certified, trainees must successfully complete the qualifying examination (Part I) followed by the certifying oral examination (Part II). Part II is a high-stakes examination administered by board-certified cardiothoracic surgeons from across the country. The purpose of the examination is to evaluate the applicants' workup, planning, and approach to patient care pre-, intra-, and postoperatively. The examination covers the disciplines of thoracic, adult cardiac, and congenital cardiac surgery. Part II is challenging for applicants due to a number of factors: the large body of material to master, the pressure and context of the examination, knowledge of previously low pass rates, the duration of time since completion of training to time of examination (including I-6 graduates whom have never experienced an oral exam situation), and the challenge of presenting clinical and management decisions in a clear and concise manner while subject to time restrictions and in the presence of mature colleagues.

The ABTS-reported pass rate of the certifying examination in 2017 was 84%. The pass rate has varied widely over the years, with a reported pass rate of only 70% in 2012. Trainees knowledge of variable pass rates may contribute to anxiety surrounding the examination. Trainees may feel underprepared for this portion of the board-certification process, as many have never experienced a surgical board-sponsored oral examination in cardiothoracic surgery.^4^

Formalized test preparation for the qualifying examination is limited. Currently, there are no ABTS-sponsored resources to prepare for the qualifying component of board certification. As a result, many trainees elect to participate in a private “oral board preparation” course the week before the examination. Participation in this private preparatory course is not only financially costly to the trainee, costing between $1000 and $1500, but also requires time away from work and home. Private board preparation courses introduce variability and bias into our trainees' ability to prepare for the examination due to socioeconomic and time constraints.

Mock oral examinations (MOEs) have been shown to be useful to trainees as well as to their training programs.^5^ If evaluation forms are used effectively, the trainees' performance on MOE can easily identify areas of clinical weakness and need for improvement. In addition, MOE allows for faculty members to evaluate the process whereby the examinee thinks through common cardiothoracic case scenarios. With thoughtful reflection, a training program can make needed educational changes to provide their trainees with a more robust experience to bolster their educational efforts and help to prepare them for the real examination.^5^^,^^6^

The ACGME and ABTS require that 65% of program graduates from the preceding 5 years must pass Part I and Part II upon the first attempt. This requirement incentivizes both trainees and training programs to create an educational environment that prepares program graduates to be successful on both the qualifying and certifying examination to minimize the risk of losing accreditation.

Within cardiothoracic surgery, single institutional experiences have been published surrounding curriculum development for MOEs. In many cases, however, these curricula are limited by the examination being “low stakes,” with faculty members examining trainees from their home institution, not affording the ability to simulate a more “high stakes MOE” experience.^1^^,^^7^

The coronavirus disease 2019 (COVID-19) pandemic has highlighted the ability for us as cardiothoracic surgeons to communicate and collaborate via Internet platforms like Zoom. In doing so, we have the enhanced capability to bring together faculty and trainees from across the country.^8^ Multi-institutional MOEs have been performed in major metropolitan areas such as New York and Boston for a number of years; however, due to small amounts of programs and trainees in specific geographic locations such as the Midwest, the scope and reach of collaborative cardiothoracic MOE can be broadened with the use technology. As a result, we hypothesized that creating a rigorously executed, remotely-administered high-stakes environment MOE that paired faculty with trainees from different institutions would provide a more realistic simulation and an important educational experience. Here, we report our findings and results of our pilot study evaluating the first ever virtual collaborative multi-institutional collaborative mock oral (mICMO) examination for cardiothoracic surgery.

## Methods

### Study Population

On May 27, 2020, 14 cardiothoracic surgery trainees across 6 training institutions participated in mICMO as examinees. All had previously completed general surgery residency at ACGME-accredited institutions. Fourteen cardiothoracic surgery faculty members across 6 training institutions participated in mICMO as examiners. Faculty were excluded from participation if they were involved in the ABTS examinations in any capacity as examiners or members of leadership. This study was undertaken after approval by the University of Wisconsin Madison institutional review board with a waiver of individual informed consent.

### Development of the Web-Based MOE Format

As a result of the COVID-19 pandemic, many surgeons and educators have become familiar with the use of the Zoom (San Jose, Calif) platform for conducting meetings and interviews. With the use of Zoom Breakout Rooms, separate “examination rooms” were created consisting of 1 cardiac faculty member and 1 thoracic faculty member from each of the 6 participating institutions. Each faculty pair then examined one trainee per session for 2 sessions, each lasting 45 minutes in which 2 cardiac scenarios and 2 thoracic scenarios were executed. The trainee was unknown to both of the examiners.

### Development of the MOE

Participating faculty experts, all of whom are board-certified or board-eligible cardiothoracic surgeons, were involved in the collaborative creation of the question stems. These faculty members are all in active clinical practice and participate in the education of residents at ACGME-accredited cardiothoracic training programs. Question-stem construction was conducted via a Zoom meeting during which all questions were discussed and modified for clarity and completeness. Following the virtual meeting, a document was circulated to participating faculty for additional editing. Once all faculty were in agreement regarding the 4 questions to be administered for the pilot mICMO, a final question bank was provided to all faculty examiners. Per ABTS policy, no faculty involved with administering the qualifying Part II examination was permitted to participate in mICMO development or administration.

### Administration of mICMO

Before the examination date, faculty and trainees were e-mailed specific instructions regarding the time of the examination as well as the format. Faculty were provided the finalized question stems and prompts, their faculty room partner, and the names and institutional affiliations of the trainees which they would examine. Faculty were also provided a link to a standardized trainee evaluation form and a post-examination survey for completion. A reminder e-mail was sent to both faculty and trainees 24 hours before the scheduled examination, which included the time of examination, as well as the Zoom link for log-in purposes.

On the day of the examination, examiners and examinees logged into the secure virtual link. Seven zoom “breakout” rooms were created with the preidentified faculty pairs. To accommodate all trainees, each faculty team examined 2 trainees. Four scenarios were given in each room (2 cardiac and 2 thoracic) and the time allotted per room was 45 minutes. The scenarios were identical and delivered in the same manner to trainees in both the first and second session. Trainees progressed thru each scenario with a 7-minute time limit to most accurately simulate the ABTS qualifying examination. At the conclusion of the 4 scenarios, trainees were provided verbal feedback from the faculty examiners. The trainees then left the rooms. The second session proceeded in an identical fashion with the second trainee.

For each question asked, both examiners in the faculty room scored the trainee on the following scale: pass, borderline pass, fail, or critical fail via an electronic evaluation form. In addition to the aforementioned score, faculty were able to provide written formal feedback on the trainees' performance for each question. Evaluations were then compiled by the director of mICMO (A.G.F.) and distributed directly to the examinees as well as each of the examinees home institution residency program coordinators. In doing so, the evaluations can be used by each program for the trainees mandatory ACGME Clinical Competency Committee meeting to discuss trainees educational progress. Videos were captured with consent to facilitate additional feedback on examination style.

Following the completion of the examination experience, an electronic link was sent to all faculty and trainees for them to complete a postexamination survey to gain further knowledge and insight about the experience.

## Results

This pilot study included faculty and trainees from 6 institutions (University of Wisconsin, Vanderbilt University, Case Western Reserve University, Cedars Sinai Medical Center, MD Anderson, and Memorial Sloan Kettering). Time was protected for both faculty and trainees to participate in this experience. The reported results are compiled from the electronic survey which was sent to all trainee and faculty participants at the conclusion of the pilot mICMO experience. Responses were anonymous and deidentified. At the end of the survey both trainees and faculty members had the opportunity to provide short answer feedback.

### Demographics of Trainees

Of the 14 trainees who participated, 12 are currently in traditional cardiothoracic surgery training programs, with 2 being graduates of an ACGME traditional cardiothoracic training program, currently pursuing an advanced fellowship experience. With respect to post-graduate year, 50% (n = 7) PGY-6, 14% (n = 2) PGY-7, 7% (n = 1) PGY-9, and 28% (n = 4) PGY-10.

One half of the participants had previously participated in cardiothoracic MOE. While not all trainees have previously participated in an MOE for cardiothoracic surgery, each of their training programs provides an MOE experience for trainees. When questioned about the frequency of MOE at their home institutions, 57% (n = 8) are annually, 35% (n = 5) are biannually, 7% (n = 1) are more than 4 times per year. In total, 78.6% (n = 11) have taken the American Board of Surgery (ABS) General Surgery oral board examination, whereas 21.4% (n = 3) have not.

### Utility of mICMO for Trainees

**Figure 1:**
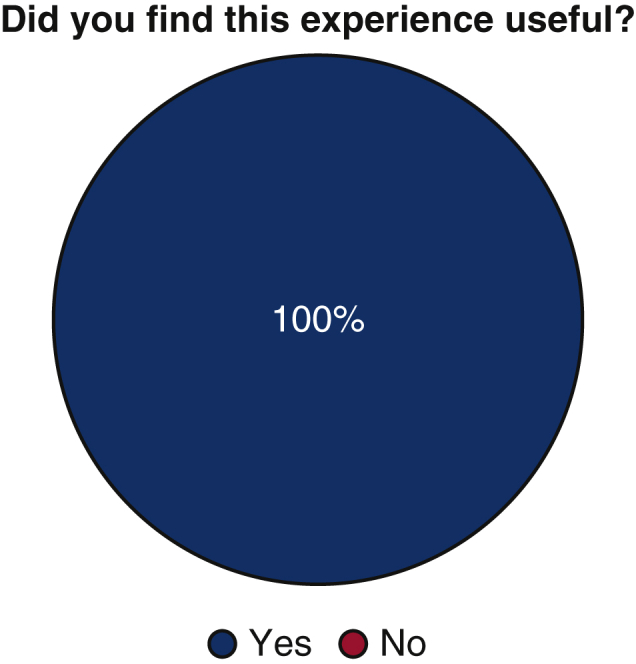
Utility of mICMO examination for trainees.

**Figure 2:**
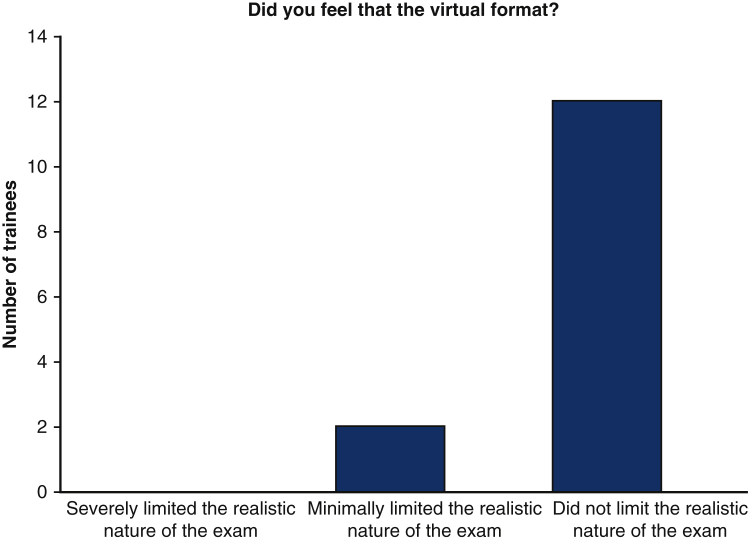
Perceived limitations of the Zoom virtual platform for mICMO examination.

100% (n = 14) of trainees responded that they found them ICMO to be useful (Figure 1). With respect to the Zoom platform virtual experience, 57.1% (n = 8) felt that the virtual experience did not limit the realistic nature of the examination, whereas 42.9% (n = 6) found the virtual experience to minimally limit the realistic nature of the examination. No trainee found the virtual experience severely limiting (Figure 2).

**Figure 3:**
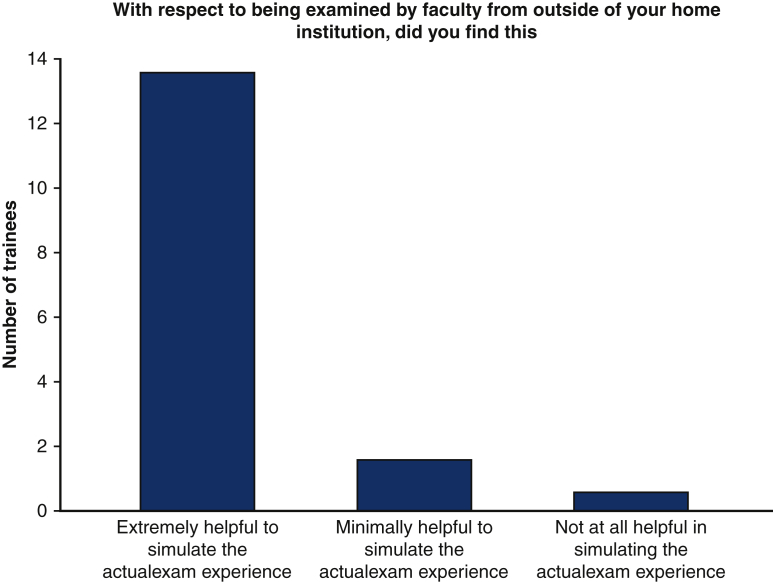
Importance of being examined by faculty outside of home institution.

**Figure 4:**
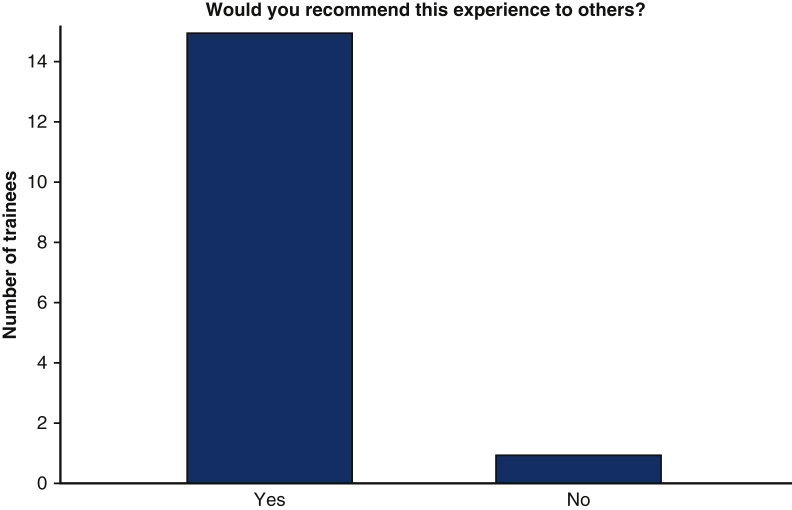
Would you recommend this experience to your peers?

When asked about the importance of being examined by faculty from outside of the trainees' home institutions, 92.9% (n = 13) found this aspect of mICMO to be extremely helpful to simulate the actual examination (Figure 3). Similarly, 92.9% (n = 13) of trainees who participated would recommend mICMO to others (Figure 4).

### Invited Trainee Comments

At the end of the trainee survey, space was provided for short answer additional comments. Representative remarks were resounding in their positive feedback for the pilot mICMO experience. Trainees remarked that mICMO was the most useful teaching tool they have ever participated in, and that it was an exceptionally helpful experience. The virtual nature of the experience was more than outweighed by the benefit of performing the examination with unfamiliar faculty members.

### Demographics of Faculty

Of the faculty members that participated, 50% of faculty specialize in thoracic surgery, 41.7% in adult cardiac surgery, and 8.3% in congenital cardiac surgery. Of the participating faculty members, 16.7% of faculty have been ABTS certified for less than one year, 41.7% between 1-5 years, 8.3% 5-10 years, and 16.7% greater than 10 years. Two faculty members are in process of becoming ABTS certified.

### Utility of mICMO for Faculty

**Figure 5:**
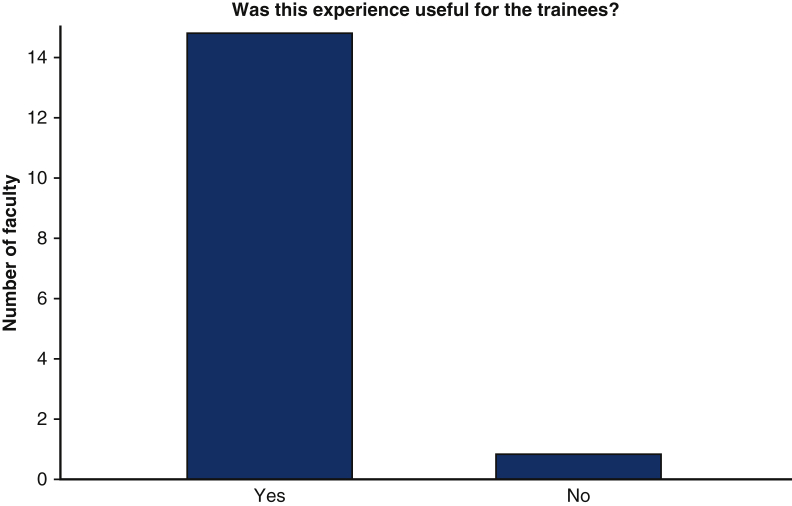
Utility of mICMO examination for faculty members. *mICMO*, Multi-institutional collaborative mock oral.

**Figure 6:**
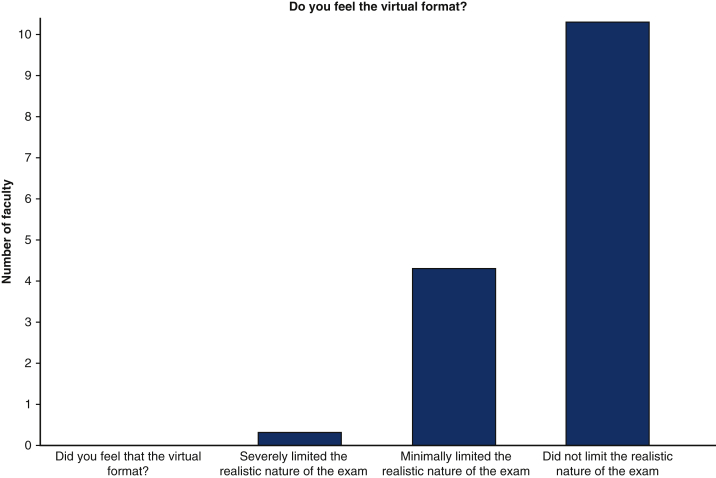
Faculty perceived limitations of Zoom virtual platform for mICMO examination.

100% of examiners found the experience useful for the trainees, with majority of faculty feeling that the virtual format did not limit the realistic nature of the examination (Figures 5 and 6). In total, 100% of faculty who participated indicated that they would be willing to participate in another mICMO experience. 100% of faculty felt comfortable administering the examination in a virtual format with the Zoom platform.

### Invited Faculty Comments

Faculty found the pilot mICMO experience to be useful for their trainees and an important educational tool. Feedback was solicited by way of survey, with comments including suggestions for a timer function to keep strict to the 7-minute requirement, a desire to increase the frequency of mICMO, perhaps quarterly, as having examiners from other centers was recognized as a huge strength. Finally, faculty commented that having additional questions and firm requirements for grading would be important.

## Discussion

The ABTS Certifying Examination (CE) is a high-stakes, broadly focused, examination conducted in an unfamiliar testing environment and setting. Due to these issues, combined with lack of rigorously tested and implemented educational resources for examination preparation, many trainees express anxiety and find preparing for the examination challenging.

Within the general surgery literature, the utility of institutional collaboration surrounding MOE has been studied. Multi-institutional MOE have been shown to improve the first-time pass rate on the ABSCE.^9^ Fingeret and colleagues^10^ retrospectively analyzed residents from 3 academic medical centers who participated in a regional MOE between 2011 and 2014. After studying 88 residents, multivariable logistic regression identified participation in multiple multi-institutional MOE being associated with first-time pass rate of ABSCE.^10^

Within our specialty, single institutional experiences surrounding formalized MOE have been developed and studied, which have found to be beneficial to our trainees. In a single institutional experience at MD Anderson, Corsini and colleagues^11^ retrospectively reviewed their twice-yearly MOE experience over a 5-year period. The authors found that the pass rates for sequential MOEs demonstrated a gradual improvement in trainee performance, and when these individuals took the ABTS certifying examination, the pass rate was 100%. This study, although small and from a single institution, provides evidence supporting the importance of MOE to our trainees' education,^11^

The COVID-19 pandemic has caused physicians and training programs to rapidly change practice due to the need for social distance, safer at-home mandates, hospital redistributions of resources, and a shift to electronic platforms for clinical consultations and educational sessions. A silver lining of the pandemic is our new comfort with communicating virtually. From an educational standpoint, with this comfort comes the ability to easily interface with institutions, networks, and individuals with whom collaboration would previously feel uncomfortable due to geographic limitations and siloed training programs.^8^

Creating a virtual high-stakes MOE provides a unique educational opportunity for our cardiothoracic trainees, capitalizing on the ability of electronic platforms to cross geographic and institutional boundaries. Compared with general surgery, the field of cardiothoracic surgery is small, and establishing a multi-institutional collaborative in person experience based on geographic location as Fingeret reported on would be challenging. As a result, mICMO was developed across 6 institutions.

The results of the pilot mICMO experience are encouraging. All trainees and faculty surveyed found the experience to be useful, and nearly all trainees would recommend mICMO to their peers, citing the opportunity to be examined by faculty from outside of their home institution as extremely helpful in simulating the actual examination experience. This finding is important, as trainees find the qualifying examination to be psychologically stressful and therefore difficult to deliver answers in a thoughtful, clear and concise manner. The mICMO experience has allowed them the opportunity to simulate this high-stakes environment in a low-stakes situation. From a faculty standpoint, the majority felt that the virtual format did not limit the realistic nature of the examination, and participating faculty indicated that they would be willing to participate in another mICMO examination.

These findings build enthusiasm and underscore the need and desire of both our faculty and trainees to participate in collaborative educational efforts across institutions. This study builds on previous multi-institutional cardiothoracic surgical education efforts under the umbrella of Thoracic Educational Cooperative Group (TECoG), an organization founded on the premise that advances in thoracic surgical education could be made by capitalizing on the educational expertise across training programs, as well as using multiple programs to increase the number of subjects, given the typically small numbers of cardiothoracic surgical trainees within a single program.^12^^,^^13^

Moving forward, it will be important to fully quantify the impact of mICMO on the trainees who have participated based on pass rates on the certifying ABTS examination. Due to varying postgraduate years of the inaugural group of participants, these data will not be available for some time. However, a surrogate marker to investigate will be trainees' performance on subsequent mICMO examinations. Areas of minor improvement, such as providing a timing device and the ability to project images and videos for use in the question stem, are being explored and implemented.

Assuming continued interest and increased participation in mICMO throughout the country due to the pilot being overwhelmingly positive, the onus will be on faculty participants and key mICMO leaders to create and maintain a comprehensive set of questions that are banked and stored for use. This question bank will be created with a clear set of coincident metrics for a trainees' performance on the mICMO examination. In addition, continued data collection in the form of trainee performance, numbers of mICMO examinations taken, postgraduate year in training, and pass/fail status of the ABTS qualifying examination will continue to be collected to objectively quantify the impact of mICMO.

Using the Zoom electronic virtual platform for mICMO has additional educational advantages that have not yet been fully explored. The ability to film each room and save the recordings can allow trainees to review their performance privately, affording critical self-assessment on the delivery, cadence, and quality of their examination. This is important as the certifying examination is not only about testing the trainees fund of knowledge but also dependent upon their ability to succinctly deliver verbal answers in a high-stakes environment. In addition, the ability to review each room following the examination can allow for quality control and improved examiner performance in order to provide the most realistic simulation of the certifying examination. Finally, an opportunity exists for independent review of the performance of both the examiner and examinee by an unaffiliated faculty member. This type of independent external review can serve an important role in standardizing the question stem delivery and examination skills of faculty members, and more accurately scoring the examinees.

Due to the enthusiasm on behalf of both trainees and faculty members who participated in the pilot mICMO experience, an obvious next step will be to expand access to mICMO to more trainees and faculty members across the country. This type of large, broad, collaborative effort may be enhanced by partnering with the TECoG component of the Thoracic Surgery Directors Association, as the goals of mICMO and TECoG are well in alignment.

There are certain limitations to this study. While it represents a collaborative effort across multiple institutions, the number of participants is still relatively small and due to the retrospective nature is subject to the associated bias. The trainees included in this pilot experience are all traditionally trained fellows. As a result, how to implement this program among I-6 trainees was not studied. The I-6 group represents a unique subset of cardiothoracic trainees based on the wide variety of clinical experience that the trainee will have based on their year in the program. Further considerations must be taken into account as mICMO grows to include this subset of individuals. With only one pilot experience with which to report, the results are largely descriptive and subjective. As mICMO continues to grow, additional data will be collected, furthering our collective knowledge on the impact of multi-institutional collaboration in trainee preparation for the ABTS certifying examination.

## Conclusions

MOE have been shown in cardiothoracic surgical literature to be beneficial to our trainees based on single institutional experiences. Here, we report the results of the pilot mICMO experience, a multi-institutional collaboration across 6 cardiothoracic training programs in the United States. Faculty and trainees alike found the experience to be effective, educationally beneficial/useful, and productive in simulating the ABTS certifying examination. These results support the continued use of mICMO for our cardiothoracic trainees and encourage continued expansion and collaboration with additional institutions across the country.

### Conflict of Interest Statement

The authors reported no conflicts of interest.

The *Journal* policy requires editors and reviewers to disclose conflicts of interest and to decline handling or reviewing manuscripts for which they may have a conflict of interest. The editors and reviewers of this article have no conflicts of interest.
